# Germline polymorphisms of the NOD2 pathway may predict the effectiveness of radioiodine in differentiated thyroid cancer treatment

**DOI:** 10.1007/s40618-024-02389-0

**Published:** 2024-05-16

**Authors:** M. Borowczyk, M. Kaczmarek-Ryś, S. Hryhorowicz, M. Sypniewski, D. Filipowicz, P. Dobosz, M. Oszywa, M. Ruchała, K. Ziemnicka

**Affiliations:** 1 Department of Endocrinology, Metabolism and Internal Medicine, Poznan University of Medical Sciences, 49 Przybyszewskiego Street, 60-355 Poznan, Poland; 2 Institute of Human Genetics, Polish Academy of Sciences, Poznan, Poland; 3 University Cancer Diagnostic Center, Poznan University of Medical Sciences, Poznan, Poland; 4 Institute of Genetics and Biotechnology, Faculty of Biology, University of Warsaw, Warsaw, Poland

**Keywords:** Chronic lymphocytic thyroiditis, Differentiated thyroid cancer, Radioiodine treatment, Single nucleotide polymorphisms, Thyroglobulin

## Abstract

**Purpose:**

Differentiated thyroid cancer (DTC) presents a complex clinical challenge, especially in patients with distant metastases and resistance to standard treatments. This study aimed to investigate the influence of specific genes and their germline single nucleotide polymorphisms (SNPs) linked to both inflammatory processes and other neoplasms on the clinical and pathological characteristics of DTC, particularly their potential impact on radioiodine (RAI) treatment efficacy.

**Methods:**

This retrospective analysis involved a cohort of 646 patients diagnosed with DTC after thyroidectomy. Study covering 1998–2014, updated in 2023, included 567 women and 79 men (median age: 49; range: 7–83). SNP selection targeted functional significance, while mutational status was assessed by pyrosequencing for comprehensive characterization. Patient genetic profiles were assessed for associations with disease characteristics, RAI response, and cancer pathology.

**Results:**

Significant correlations emerged between certain SNPs and DTC features. Notably, the *NOD2* c.802 T > C variant (rs2066842) was identified as a marker distinguishing between papillary thyroid cancer (PTC) and follicular thyroid cancer (FTC). Moreover, the c.802 T allele was associated with an enhanced response to RAI treatment, indicating a more substantial decrease in posttreatment stimulated thyroglobulin (sTg) concentrations. The *NFKB1A* allele c.126A (rs696) exhibited connections with lower FTC stages and a reduced probability of multifocality.

**Conclusion:**

This study explored the molecular mechanisms of particular SNPs, highlighting the role of *NOD2* in innate immunity and the stress response, and its potential impact on RAI efficacy. This research underscores the clinical promise of SNP analysis and contributes to personalized treatment strategies for DTC, emphasizing the relevance of genetic factors in cancer progression and treatment outcomes.

## Introduction

Differentiated thyroid cancer (DTC) is the prevailing endocrine malignancy [1] and is predominantly treated with surgery and radioiodine [2]. Despite its generally promising clinical trajectory, patients presenting with distant metastases encounter a significant challenge, with a five-year cancer-specific survival rate of merely 40% [3, 4] This population often develops resistance to radioiodine, posing a substantial hurdle in managing their condition [5].

Differentiated thyroid cancer (DTC) has been shown to potentially be associated with inflammation, particularly chronic lymphocytic thyroiditis (CLT) [5]. Several studies have indicated a potential link between the presence of CLT and an increased risk of developing DTC [6–8]. However, they also showed a protective role of CLT in preventing the spread of DTC by limiting tumor growth to the primary site [9], possibly mediated by the cancer microenvironment [10, 11] and cellular cytotoxic and humoral immune reactions [6, 12].

Inflammation within the thyroid gland, as observed in CLT, may create an environment conducive to the initiation or progression of thyroid cancer [5, 13, 14]. However, the exact relationship between inflammation and the development of DTC is complex and requires further investigation to determine the mechanisms involved and the extent of their influence on cancer progression [15, 16]. Moreover, data on the impact of the CLT on the clinical and pathological parameters of DTC are ambiguous [15–21].

Advancements in our understanding of differentiated thyroid cancer (DTC) have been propelled by innovative genome-analyzing techniques such as high-throughput next-generation sequencing (NGS) [22, 23]. These methods have revealed shared molecular pathways between DTC and various other malignancies [24]. However, the interconnections among differentiated thyroid cancer, inflammation, and the pathogenesis of other malignancies remain unclear and warrant further investigation [25, 26].

Although strides have been made in understanding how tumor-specific genetic changes can predict outcomes, the accuracy of forecasting outcomes for DTC patients remains significantly constrained by its low specificity [27]. This limitation predominantly affects the care of individuals with nonadvanced disease, encompassing a diverse group of patients with DTC, many of whom typically experience remission after thyroid ablation (surgery with or without radioiodine) but might encounter recurrence later [2, 28]. The progression of cancer could hinge more on a patient’s genetic background than on their environmental influences, hinting at the possible involvement of inherited traits in the progression of cancer [29, 30]. Furthermore, for ascertaining germline mutational status, the acquisition of a patient’s blood sample is notably more convenient and accessible than obtaining tumor samples for determining somatic mutational status. Therefore, germline mutational status may help in describing the prognosis of this disease and the best method of treatment.

Consequently, this study primarily aimed to assess the influence of specific genes and their germline single nucleotide polymorphisms (SNPs) linked to both inflammatory processes and other neoplasms on the clinical and pathological characteristics of DTC in a homogenous patient population. The secondary objective was to describe the potential impact of these agents on the efficacy of radioiodine treatment in managing DTC.

## Patients and methods

### Patient characteristics

We retrospectively analyzed 646 patients diagnosed with thyroid cancer at the single endocrinological center in Poznan according to the fifth edition of the Classification of Endocrine and Neuroendocrine Tumors released by the World Health Organization (WHO). The group consisted of 567 women and 79 men who were Caucasian, with a median age at diagnosis of 49 years and ranging from 7 to 83 years. Patients diagnosed with differentiated thyroid cancer after thyroidectomy were enrolled in the study. The exclusion criterion was the lack of a patient's histopathological description of DTC. The date of diagnosis was set as the date of thyroidectomy.

The analysis covers the data collected between 1998 and 2014 and updated in March 2023. The Bioethical Committee of Poznan University of Medical Sciences approved the study (approval no. 629/07 from June 2007), which was conducted in accordance with the Declaration of Helsinki.

### Histopathological, laboratory and clinical data

CLT diagnosis was confirmed through a detailed examination of tissue obtained from thyroidectomy at the time of DTC diagnosis. This examination revealed the widespread infiltration of lymphocytes and oxyphilic cells and the development of both primary and secondary lymphoid follicles [31, 32]. We considered Hashimoto’s thyroiditis and the expansion of individual lymphoid follicles to be CLTs [33]. To avoid biases, we defined CLT considering the histopathological examination (infiltration of lymphocytes) without the autoimmune pattern (antibody positivity).

Tumors were categorized as multifocal when two or more distinct areas were identified. In the case of multifocality, the tumor size was determined by the largest focus. The staging procedures adhered to the American Joint Committee on Cancer TNM staging system (8th Edition) [34].

The molecular information was integrated into the clinical context by correlating it with the histopathological analysis of specimens obtained from thyroidectomy, along with the available clinical data. These included details about the patients and samples, such as age at diagnosis, sex, tumor size, the presence of multifocality (when two or more distinct areas were detected), extrathyroidal extension, evidence of chronic lymphocytic thyroiditis in histopathological assessments, histopathological staging (pTNM) following the 8th edition of the tumor-node-metastasis (TNM) classification [2, 34], concentrations of stimulated thyroglobulin (sTg), antithyroglobulin antibodies (aTg), anti-thyroid peroxidase antibodies (aTPO), and thyrotropin (TSH), and the difference between sTg at first qualification to radioiodine (RAI) treatment and sTg at one year after RAI administration (so-called delta sTg). All sample information was anonymized to maintain patient confidentiality and privacy.

The stimulated thyroglobulin concentration was assessed by an immunoradiometric assay with the use of Brahms diagnostic kits and was determined to be credible for TSH stimulation [35] when the concentration was above 30 μIU/ml (assessed by the electrochemiluminescence method with the use of Roche diagnostic kits and the COBAS e601 analyzer from Hitachi).

All individuals examined in the present study agreed to undergo genetic testing in accordance with the requirements of the Ethical Committee of Poznań University of Medical Science (acceptance no. 629/07).

### SNP selection

The choice of candidate SNPs (refer to Table 1) was guided by their documented functional significance, if available, and their established connections with radiosensitivity or the risk of cancer, especially thyroid cancer.

**Table 1:** Single-nucleotide polymorphisms (SNPs), possible functional roles and effects on thyroid cancer

Gene	Change in DNA	Change in protein	rs number	Possible genotypes	Links with thyroid cancer (references)	Other neoplasms affected
*P53*	c.215C > G	p.Pro72Arg	rs1042522	CC CG GG	DNA damage response genes	Basal cell carcinoma, Nasopharyngeal carcinoma, Pancreas Carcinoma, Choroid plexus papilloma, Adrenocortical carcinoma, Bone osteosarcoma, Colorectal cancer, Hepatocellular carcinoma, Familial cancer of breast, Hereditary cancer-predisposing syndrome, Hereditary breast ovarian cancer
*NFKBIA*	c.126G > A	3' prime UTR variant	rs696	GG GA AA	Alters NF-κB activity	Gastric cardia cancer Prostate cancer Multiple myeloma Colorectal cancer
*CHEK2*	c.470 T > C	p.Ile157Thr	rs17879961	TT TC CC	Defective in its ability to bind p53, BRCA1 and Cdc25A proteins	Prostate cancer Breast cancer Ovarian cancer Colon cancer Kidney cancer Adrenal cortex carcinoma, Gastrointestinal carcinoma
*CHEK2*	c.433G > A	p.Arg145Trp	rs137853007	GG GA AA	not specified	Familial breast cancer Hereditary cancer-predisposing syndrome Ovarian cancer Stomach cancer
*ERCC2*	c.171G > C	3' prime UTR variant	rs3916891	GG GC CC	not specified	not specified
*ERCC2*	c.2251 T > G	p.Lys751Gln	rs13181	TT GT GG	DNA repair pathway No relationship	Head and neck squamous cell carcinomas Colon cancer Cutaneous melanoma Ovarian cancer
*NOD2*	c.2140C > T	p.Arg702Trp	rs2066844	GG GA AA	not specified	not specified
*NOD2*	c.2722G > C	p.Gly908Arg	rs2066845	GG GC CC	not specified	not specified
*NOD2*	c.802C > T	p.Pro268Ser	rs2066842	GG GA AA	not specified	not specified
*NOD2*	c.3019_3020insC	p.Leu1007fsinsC	rs2066847	Wild type insC insC/insC	not specified	not specified
*TNFA*	g.4752G > A	5' TNFA flanking sequence variant	rs361525	GG GA AA	not specified	not specified
*CCND1*	c.669C > T	p.Phe223 =	rs3862792	CC TC TT	not specified	not specified
*CCND1*	c.723G > A	p.Pro241 =	rs9344	CC TC TT	modulates the frequency of the alternative mRNA folding Differentiated thyroid cancer	laryngeal cancer colon cancer Breast cancer salivary gland cancer

*NA*—not applicable

The first step was to define the list of SNP, which already been linked to increased risk of developing cancer, especially DTC and/or to radioiodine sensitivity. Our search strategy included Medical Subject Headings terms and keywords: “Single Nucleotide Polymorphism” AND “thyroid cancer” OR “cancer” OR “radioiodine”. Reference lists of all the selected articles, previous meta‑analyses, and reviews were hand‑searched for any additional articles. We included studies, regardless of their sample size, that investigated the association between SNP and DTC occurrence or cancerogenesis or radioiodine sensitivity. We carried out a systematic review following the guidelines formulated in the Cochrane Handbook for Systematic Reviews of Interventions and the Preferred Reporting Items for Systematic Reviews and Meta‑Analyses (PRISMA) guidelines. We searched the following databases: PubMed, MEDLINE, Academic Search Complete, CINAHL Complete, CINAHL, Scopus, Cochrane, Health Source: Nursing/Academic Edition, Web of Knowledge, MasterFILE Premier, Health Source‑Consumer Edition, Agricola, Dentistry, and Oral Science Source from January 2000 up to January 2023 to find all relevant, full‑ text journal articles written in English.

Three authors (MB, MKR, SH) independently selected the publications that met the inclusion criteria specified above and extracted data for the outcomes using a standardized data extraction form. Abstracts/papers focusing on medullary thyroid cancer were excluded. The final list of SNPs was identified.

This selection process did not involve seeking tag SNPs or considering the genetic diversity within the regions where these SNPs are located. All the SNPs listed are cataloged in the public database dbSNP (Single Nucleotide Polymorphism Database) and have been validated across diverse ethnic populations. To ensure robustness in our calculations, only SNPs with a minor allele frequency (MAF) exceeding 1% were incorporated into the analysis.

### Molecular studies

Assessment of mutational status was conducted with pyrosequencing. DNA was extracted from whole peripheral blood leukocytes with guanidine isothiocyanate and phenol‒chloroform. The primers used were designed with PyroMark Assay Design Software 2.0 (Qiagen, Venlo, Limburg, Netherlands). The mutational status was assessed from the patients’ initial blood samples, acquired at the time of thyroidectomy or within a period of maximum six months from the time of diagnosis. Reactions were performed on a PSQ96 device (Pyrosequencing AB, Uppsala, Sweden) using the PyroMark Gold Q96 reagent kit (Qiagen, Venlo, Limburg, Netherlands).

To identify the genes within the CNVs, we used the UCSC database [36] and Ensemble [37]. Gene annotation and gene overlap were assigned using the human genome build 19 (hg19) and NetAffx [38]. In addition, the identified alterations were compared with data deposited in the COSMIC database [39] to look for overlaps with up-to-date known genomic cancer regions and genes.

### Statistical analysis

To estimate the effect of the variants on the thyroid cancer type (PTC vs FTC) and stage (pT1b-pT4 vs pT1a), we used a logistic regression model defined as follows:

$$y = X\beta + Zu + e$$

where y is an $ln = \frac{P}{1-P}$ with *P* being the probability of developing FTC and the probability of diagnosing the pT1a stage of FTC. *X* and *Z* are design matrices corresponding to β, a vector of nongenetic effects of age at the time of diagnosis and sex, respectively. *u* corresponds to the genetic effect of the analyzed variant, where this effect is assumed to be dominant where both homozygous and heterozygous mutations contribute equally or as additive where the variant effect increases with the number of mutant alleles.

**Fig. 1:**
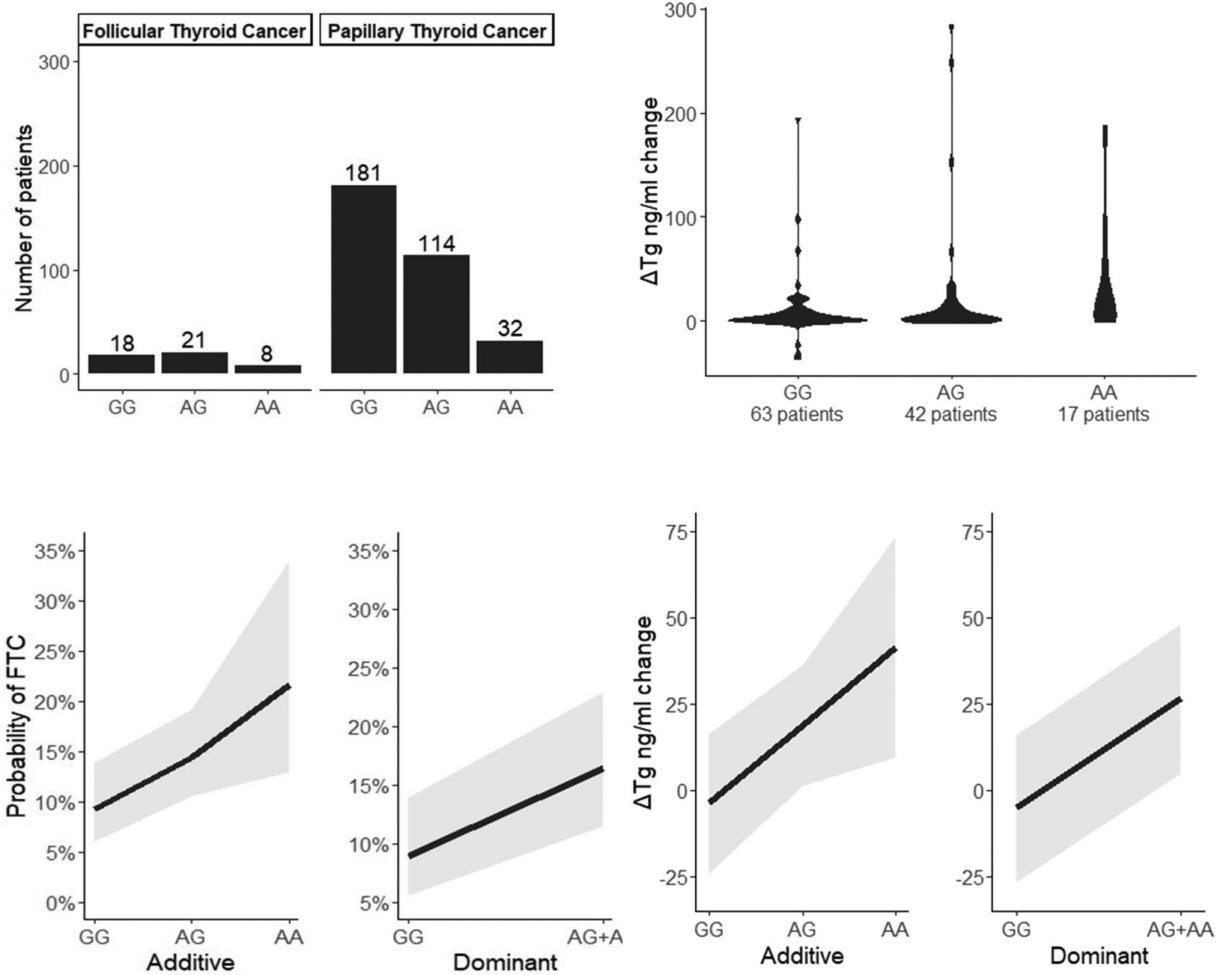
Probability of **a** FTC and **b** a decrease in sTg after RAI treatment (delta sTg) depending on the mutational status of NOD2 rs2066842

**Fig. 2:**
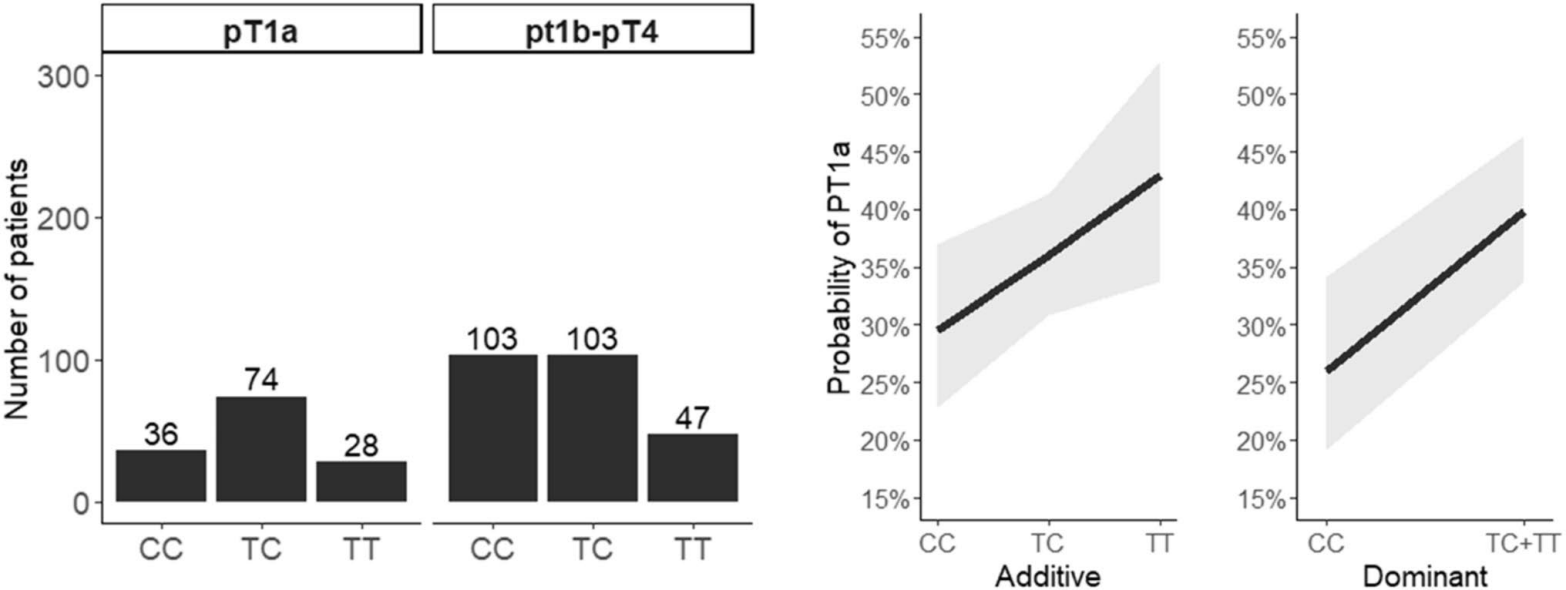
Probability of pT1a according to the *NFKB1A* rs696 mutational status

Variant effects on the delta sTg were estimated with a linear regression model, where *y* was defined as the average change in the delta sTg ng/ml. Nongenetic, and genetic effects were modeled in the same way as in the logistic regression models. Due to the large portion of missing data and the different number of samples per variant, we decided to analyze each variant separately instead of via joint analysis. All analyses were performed using a generalized linear model implemented in R, and all the visualizations were performed with the ggplot2 package [40, 41]. For easier interpretation, we estimated marginal effects for each analyzed variant [42], i.e., change in the phenotype (probability or mean) when the allele count was changed by one unit (Figs. 1, 2).

To compare differences between groups, we used the chi-square test or Fisher’s exact test (2 × 2 contingency table), as appropriate, for categorical variables. Interval data were compared with the use of the Mann‒Whitney U test with post hoc Dunn’s test since the data did not follow a normal distribution. Odds ratios (ORs) and 95% confidence intervals (95%CIs) were calculated. To compare differences between the groups for categorical variables, the chi-square test was used if the Cochrane assumptions were met; otherwise, Fisher’s exact test was used. Interval data were compared with the use of the Mann–Whitney U test, as the data were not normally distributed [43]. A *p* value of less than 0.05 was regarded as significant. Statistical analyses were performed with StatSoft Statistica v13.0, PQStat v1.6.8, and R v4.3.2 software.

## Results

A total of 634 patients with DTC were ultimately included in the study. Among them, 549 patients were diagnosed with papillary thyroid cancer (PTC), and 85 were diagnosed with follicular thyroid cancer (FTC). There was no significant difference in sex distribution or age at diagnosis between patients with PTC and those with FTC. Patient characteristics are presented in Table 2.

**Table 2:** Clinical characteristics of patients with papillary and follicular thyroid cancer

Characteristics	Papillary Thyroid Cancer N = 549	Follicular Thyroid Cancer n = 85	*P* value
Male/female, n (%)	66/479 (12.1/87.9)	11/74 (12.9/87.1)	0.93
Median age at diagnosis/NA, years (range)	49.5/59 (7–78)	49/15 (13–83)	0.43
Age group ≤ 60 years/ > 60 years/NA, n (%)	387/110/59 (69.6/19.8/10.6)	53/18/15 (61.6/20.9/17.4)	0.0034/0.94
Median length of follow-up/NA, months (range)	228/75 (132–624)	228/6 (132–516)	0.63
Multifocality/NA, n (%)	111/285 (20.4/52.3)	11/58 (12.9/68.2)	1.00
Capsule invasion/NA, n (%)	40/452 (7.34/82.9)	12/67 (14.1/78.8)	0.11
Nodal involvement/NA, n (%)	87/56 (16/10.3)	11/13 (12.9/15.3)	0.72
Mean tumor size, mm/NA (range)	13.4/265 (1–90)	28.4/51 (2–100)	$1.052 \times 1{0}^{-6}$
Tumor diameter ≤ 10 mm/NA, n (%)	127/265 (23.3/48.6)	29/51 (34.1/60)	$2.48 \times 1{0}^{-5}$
Localization in the left/right/both lobes/NA, n (%)	29/34/13/469 (5.32/6.24/2.39/86.1)	9/4/0/72 (10.6/4.71/84.7)	0.06
Chronic lymphocytic thyroiditis/NA, n (%)	60/269 (11/49.4)	7/53 (8.24/62.4)	1.00
The need for second dose of radioiodine/NA, n (%)	96/374 (17.6/68.6)	10/62 (11.8/72.9)	0.36

*NA*—not available

A total of 236 patients were diagnosed with DTC and CLT. The mean concentration of sTg was 20.74 ng/ml ± SE 59.97, the mean concentration of aTG was 53.27 IU/mL ± SE 176.94, and the mean concentration of aTPO was 150.70 IU/ml ± 295.45. The expression of any of the analyzed SNPs differed between patients with and without CLT (Table 2).

Among the identified NOD2 rs2066842 SNPs, differentiated PTC from FTC and the c.802 T allele were more common in patients with follicular thyroid cancer than in patients with papillary thyroid cancer (additive model: p = 0.02, OR = 1.65 ± SE 0.40–2.89; dominant model: p = 0.031, OR = 2.00 ± SE 0.62–3.38).

All patients who had both FTC and CLT were *NOD2* rs2066842 positive.

sTg at first hospitalization was greater in patients with CLT (mean 5.35 vs. 2.17, p = 0.003). In all patients with DTC, the *NOD2* rs2066842 allele c.802 T was associated with a greater decrease in thyroglobulin after the first radioiodine administration (delta in sTg at first qualification to RAI and sTg one year after the first administration of RAI), with 0.03 and a mean ΔTg 22.54 ± SE 12.32–32.76 ng/ml per mutant allele (c.802 T) in the additive model and a mean delta ΔTg 31.78 ± SE 17.07–46,49 ng/ml for carriers in the dominant model.

The *NFKB1A* rs696 allele c.126A was associated with a lower stage of FTC (pT1b-pT4 vs pT1a), with an additive OR = 1.02 ± SE 0.01–2.025 and a dominant OR = 1.8971 ± SE (0.631.12–3.152.61); p = 0.00613. The *NFKB1A* rs696 allele c.126A was also associated with a lower probability of multifocality (OR = 1.74 [1.02–2.97, p = 0.04]).

## Discussion

The results of our study, performed in the largest reported homogenous cohort of subjects to date, confirmed that SNPs may be involved in the clinical course of DTC. Among these polymorphisms, the *NOD2* rs2066842 polymorphism may distinguish PTC from FTC. It may also be a marker of better response to RAI in DTC patients, as it was linked with a greater decrease in sTg before and after the first RAI administration. Moreover, *NFKB1A* rs696 may predict a lower stage of FTC and a lower probability of PTC multifocality.

Critical factors influencing thyroid tumorigenesis are genetic. A major role is attributed to the activation of proto-oncogenes, wherein the basal expression level of oncogenes regulates cell growth and differentiation by participating in signal transduction to the cell nucleus. These include growth factors, receptors for growth factors, tyrosine kinases, and transcription factors.

Like the activation of oncogenes, the inactivation of tumor suppressor genes, such as *TP53*, whose function is to prevent tumorigenesis, is of great importance. Loss or inactivation of both alleles of a tumor suppressor gene leads to tumor development. Mutation of the *TP53* gene is the most known genetic alteration involved in human cancer formation. On the other hand, mutations in the *TP53* suppressor gene are associated with mutations in the *CHEK2* gene and are associated with the Li-Fraumeni syndrome (LFS) phenotype. Mutation in mutator genes that can repair mutations leading to oncogene activation or suppressor gene inactivation may also be a cause of the tumorigenesis process.

Other genetic factors may include abnormalities in growth factors and their receptors, such as tumor necrosis factor (TNF) and other cytokines. Because single nucleotide changes may result in increased protein expression, a variant of the TNF gene has also been of interest to us.

The nucleotide-binding oligomerization domain containing 2 (*NOD2*) gene, also known as *CARD1*5, is located at chromosomal region 16q21 [44]. *NOD2* is a member of the evolutionarily conserved Nod-like receptor (NLR) family and plays a crucial role in detecting elements within microbial cell walls [45, 46]. The gene is a NOD1/APAF-1 family member that encodes proteins with two caspase recruitment domains and six leucine-rich repeats (LRRs) [47]. Studies suggest its involvement in controlling both programmed cell death (apoptosis) and long-term inflammatory disorders. The protein NOD2 is primarily expressed in humans in peripheral blood leukocytes [47]. It plays a vital role in the immune response, especially involving intracellular bacterial lipopolysaccharides (LPSs), by recognizing muramyl dipeptide (MDP), which is derived from bacteria and, in this way, activating the NFκB protein [47]. Interestingly, it has been suggested that the NOD2 protein acts as a regulator of appetite by sensing MDP molecules in a subset of brain neurons: microbial MDP may reach the brain and bind and activate the NOD2 protein in inhibitory hypothalamic neurons, leading to a decrease in neuronal activity, thus regulating satiety perception and probably influencing body temperature [48].

The role of NOD2 has yet to be determined, but it seems that it might be involved in many immunological pathways. It plays an essential role as a regulator of autophagy, especially in dendritic cells, via its interaction with the ATG16L1 protein, possibly through recruiting ATG16L1 at the site of bacterial entry [49]. Studies have also shown that NOD2 activation in the small intestine crypt contributes to intestinal stem cell survival and proper function. Hence, NOD2 may act by promoting mitophagy via its association with ATG16L1, as mentioned above [50, 51]. In addition to its important role in innate immunity, NOD2 controls the adaptive immune system by regulating helper T cells and regulatory T cells (Tregs) [50, 51]. In addition to being involved in pathogen recognition, NOD2 is involved in the endoplasmic reticulum stress response. It may act by sensing and binding to sphingosine-1-phosphate (S1P), a cytosolic metabolite generated in human cells in response to endoplasmic reticulum stress. This event usually initiates an inflammatory process leading to the activation of NF-kappa-B and MAP kinase signaling, which is very often dysregulated in cancer [50, 51].

The rs2066842 missense mutation is one of the most studied polymorphisms. It was initially found to be associated with an increased risk of Blau syndrome, as well as Crohn’s disease and ulcerative colitis [52]. It is located at coding regions and might affect the expression and function of *NOD2* by altering amino acids. For the first time, NOD2 polymorphisms were linked to the risk of colorectal cancer [53]. Subsequently, further studies revealed an association between *NOD2* polymorphisms and the risk of various cancers, including breast cancer and ovarian, endometrial, gastric, and laryngeal cancers [54]. However, the findings across individual studies varied and lacked consistency. A meta-analysis investigating overall cancer risk in relation to *NOD2* polymorphisms showed that the *NOD2* rs2066844 C/T, rs2066845 C/G, and rs2066847 (3020insC) polymorphisms might be associated with increased cancer risk. However, no significant association was observed between the *NOD2* rs2066842 C/T polymorphism and cancer risk. However, thyroid cancer was not included in the analysis [54]. Only one group has studied the role of rs2066842 in thyroid cancer, and no increase in the occurrence of this gene was detected [55].

Our study showed a difference between FTC and PTC and was even more clinically relevant. Our study showed that the *NOD2* rs2066842 polymorphism was linked to a better response to the RAI. *NOD2* rs2066842 was linked to a greater decrease in the sTg marker after the first RAI.

The sTg measurement is the cornerstone of modern management of differentiated thyroid cancer, and clinical decisions on treatment and follow-up are based on the results of such measurements [35]. Tg production occurs primarily within normal and well-differentiated malignant thyrocytes, rendering Tg an ideal “tumor marker” after elimination of both healthy and pathological tissue. The introduction of highly sensitive thyroglobulin assays has significantly enhanced analytical sensitivity and stability in measuring Tg within the lower detection range. This has substantial implications for interpreting results in contemporary clinical practice [35]. A significant correlation existed between decreasing concentrations of sTg and the disappearance of thyroid tissue and between decreasing concentrations of sTg and RAI treatment efficacy.

RAI ablation success is the main predictor of DTC overall prognosis. Therefore, markers of RAI efficacy are highly needed. According to our study, *NOD2* rs2066842 may be an easy and stable marker for the prediction of RAI efficacy.

It is not clear which mechanism, *NOD2* rs2066842, may facilitate RAI action. One possible hypothesis is that the inflammatory process in which the NOD2 pathway participates may be involved in RAI action or may be protective against the progression of DTC. RAI activity is determined by host genetic background rather than the environment [56]. Therefore, SNPs may demonstrate prognostic value in DTC. Based on our data, the SNP *NOD2* rs2066842 seems to be the most feasible candidate for determining the efficacy of RAI in DTC patients, thus affecting prognosis. Increased expression of NOD2 has been documented in various human metabolic disorders and chronic diseases linked to mitochondrial dysfunction [57]. It may manifest through direct signaling pathways, indirect modulation of cellular stress responses, or exacerbation of inflammatory processes [57]. RAI therapy in patients with DTC may increase proliferative lymphocyte responses and interferon-γ levels, demonstrating its proinflammatory function [58, 59].

The ongoing discourse revolves around the pivotal role of the cancer microenvironment. Cunha et al. proposed that the protective effect of CLT against the spread of DTC could be attributed to the involvement of various immune cells, such as CD4 + , CD8 + , CD201 + , Th17, and regulatory T cells (Tregs) [60]. Additionally, the interleukin-1 (IL-1) secreted by infiltrating lymphocytes in the CLT might exert an antitumorigenic effect by influencing the differentiation and replication of DTC cells [56, 61, 62]. Immune-mediated destruction of thyroid tissue may involve the NOD2 pathway, and our findings revealed the possible role of *NOD2* rs2066842.

The same *NOD2* polymorphism was found to be protective against multiple sclerosis and ameliorate the response to interferon-beta therapy, most likely as a part of a complex multidirectional system of genetic, immunological and environmental factors [63]. The NOD2 pathway may facilitate the action of pattern recognition receptors, which are germline-encoded host sensors that detect pathogens and play a crucial role in the proper function of the innate immune system [44].

According to the results of our study, *NOD2* rs2066842 may serve as a marker for response to RAI, complementing the established role of Tg.

We suggest the additional use of *NOD2* rs2066842 as a marker for response to RAI in patients with CLT and high concentration of aTg, where we suspect possible interference of Tg and aTg [35], potentially confounding and biasing the role of Tg as a marker of DTC. *NOD2* rs2066842 may offer superior prognostication for response to RAI compared to Tg alone and facilitate better planning of RAI treatment. In patients who are *NOD2* rs2066842-negative, it should be considered whether administering a higher activity of RAI would achieve the same effect as in *NOD2* rs2066842-positive patients. The inflammatory process mediated through the *NOD2* pathway and the activation of NF-kappa-B and MAP kinase signaling may enhance RAI efficacy. However, this hypothesis requires further investigation. In the text of our publication, we have clarified the suggested clinical role of NOD2 rs2066842 and its relationship with Tg.

Among the analyzed SNPs, *NFKB1A* rs696 may predict a lower stage of FTC and a lower probability of PTC multifocality. In inflammation, nuclear factor kappa B (NFKB) potentially exerts an oncogenic influence by fostering the proliferation and survival of numerous solid tumors [64]. Its protective role in DTC is consistent with the finding from the meta-analysis of Zhang et al. that *NFKB1A* rs696 seems to be associated with decreased susceptibility to cancer, especially Hodgkin lymphoma [65].

The nuclear factor-κB (NF-κB) gene, *NFKB1A*, resides on chromosome 14q13. The polymorphic variants within this gene rs2233406, rs3138053, and rs696 are positioned in regions that bind to CCAAT/enhancer binding protein and GATA binding protein 2. These variations potentially modulate the expression of IκBα, consequently impacting the activation of NF-κB. Moreover, these polymorphisms (rs2233406, rs3138053, and rs696) are directly associated with processes such as apoptosis, irregular immune cell maturation, and slowed cell growth [65, 66]. NF-κB belongs to a family of transcription factors that play crucial roles in inflammatory and immune reactions [67]. The protective effect of NFKB1A rs696 against higher stages of FTC and multifocality may be involved in these reactions.

### Limitations of the study

Our findings are affected by a factor that restricts their interpretation: selection bias. It is crucial to acknowledge this influence before drawing any conclusions. To reduce bias in selection and control for external factors, our study group primarily comprises a homogenous population (with just 4.7% nonethnic Poles within Poland). This places our group in the seventh position among 159 countries worldwide and third in Europe in terms of homogeneity [68].

Another limitation was the difference in methods used for sTg measurement (as different assays have been used in recent years [35]); however, every patient was a control for themselves, and only the difference in sTg concentration before and after RAI was considered significant in our study.

Additionally, there was a notable difference in the prevalence of patients diagnosed with PTC compared to those diagnosed with FTC in the study group. However, this disproportion is reflective of the respective ratios of these diagnoses within the general population [28].

There were missing data in our study, including genetic analysis of SNPs, particular parameters of tumor histopathology, and particular measurements of thyroglobulin. To address these issues, we excluded missing data from the analysis by pairwise deletion (we used available data for each specific analysis).

Acknowledging the limitations of this study, it is pertinent to note that SNP analysis may be considered somewhat outdated in the context of the multiomics era. Consequently, conducting research involving genome sequencing is a more advanced and beneficial approach. However, we decided to analyze SNPs because of the ease of their performance in laboratories and the relatively low price of these methods, which make it possible to perform these tests on all patients in many countries; additionally, these methods are reimbursed by national insurance systems, which is still not true for multiomics. Due to its clinical utility and cost-effectiveness, SNP analysis has also been used to study thyroid cancer [55, 69, 70].

## Conclusions

Our research identified possible stable and easily accessible prognostic markers in DTC patients. Moreover, SNPs represent a promising tool for empowering prognostic stratification of DTC and assessing the probability of RAI efficacy.

## Data Availability

The datasets generated during and/or analyzed during the current study are available from the corresponding author upon reasonable request.
